# How, When and Why People Seek Health Information Online: Qualitative Study in Hong Kong

**DOI:** 10.2196/ijmr.7000

**Published:** 2017-12-12

**Authors:** Joanna TW Chu, Man Ping Wang, Chen Shen, Kasisomayajula Viswanath, Tai Hing Lam, Sophia Siu Chee Chan

**Affiliations:** ^1^ National Institute for Health Innovation, School of Population Health Faculty of Medical and Health Sciences The University of Auckland Auckland New Zealand; ^2^ School of Nursing The University of Hong Kong Hong Kong China (Hong Kong); ^3^ School of Public Health The University of Hong Kong Hong Kong China (Hong Kong); ^4^ Center for Community-Based Research Dana-Farber Cancer Institute Boston, MA United States; ^5^ Department of Social and Behavioral Sciences Harvard TH Chan School of Public Health Cambridge, MA United States

**Keywords:** Internet, information seeking behavior, consumer health information, focus groups

## Abstract

**Background:**

The Internet has become an established source for health information. The number of individuals using the Internet to search for health information, ranging from healthy lifestyle advice to treatment and diseases, continues to grow. Scholars have emphasized the need to give greater voice and influence to health consumers. Hong Kong, being one of the most technologically advanced and connected cities in the world, has one of the highest Internet penetration rates in the world. Given the dearth of research in an Asian context, Hong Kong is an excellent platform to study individuals’ perceptions (eg, benefits and limitations on seeking health information online and how the information is used) on health information seeking.

**Objective:**

The aim of this paper was to study individuals’ perceptions on health information seeking and to document their Internet information–seeking behaviors.

**Methods:**

Five focus groups (n=49) were conducted from November 2015 to January 2016 with individuals across different age groups (18 years or above). Focus group contents were audiotaped, transcribed, and analyzed using thematic analysis techniques.

**Results:**

Older (55+ years) and less educated respondents were less likely to use the Internet to search for health information. Among individuals who obtained health information via the Internet, regardless of the severity of the health issue, the Internet was always the first source for information. Limited doctor consultation time and barriers to accessing professional health services were the main reasons for using the Internet. Convenience and coverage were regarded as the main advantages, whereas credibility and trustworthiness of health information were noted as limitations. The use of Web-based health information varied among individuals; hence, the implications on the doctor-patient relationship were mixed.

**Conclusions:**

The prevalent and increasing use of the Internet for health information seeking suggests the need for health care professionals to understand how it can be optimally utilized to improve health outcomes. Strategies for communicating and disseminating credible health information in a form that users can understand and use are essential. Due to the rapid technological and related behavioral changes, online health information seeking and its effects need to be closely monitored.

## Introduction

Hong Kong is one of the most technologically advanced and connected cities in the world. According to the Hong Kong Monthly Digest of Statistics (HKMDS), 2013, the estimated number of Internet users was 5.751 million in a population size of approximately 7.188 million. Internet access is available almost everywhere via broadband and Wi-Fi (39,796 public Wi-Fi hotspots in the city; Office of the Telecommunications Authority, 2015). According to the Census and Statistics Department (CSD) Hong Kong, 2014, more than 80% of households have personal computers connected to the Internet, with broadband penetration rates among the highest in the world. Ninety-six percent of mobile phone users access the Internet on a daily basis. The most commonly cited purpose of using the Internet is information searching (HKMDS, 2013). Acquiring health information from the Internet is also increasingly prevalent [1]. Individuals can obtain a wide range of information from healthy lifestyle advice to treatment and diseases [2,3].

Health information seeking relates to the ways in which individuals obtain information, including information about their health, health promotion activities, risks to one’s health, and illness [4]. Health information accessed via the Internet has enabled individuals to become more active collaborators in their own health [5]. The breadth and nature of health information obtained influences individual’s knowledge, beliefs, and attitudes toward a specific health behavior [6]. The Internet as a medium has the capacity to help change and promote health behaviors [7,8]; yet, the quality of the information varies widely [9-11]. The anonymity of content publishers and low rigor in monitoring and filtering Web-based content are some of the reported challenges from the abundance of inaccurate or misleading information [11,12]. Nonetheless, this shift toward individuals becoming more informed and empowered in managing their own health have implications on the ways they interact with professionals and the health care system [5].

Internationally, increasing studies have been conducted into understanding individuals’ perceptions on Internet health information seeking [9,13-15]. Nonetheless, there is a dearth of research on users’ perceptions on the benefits and limitations on seeking health information via the Internet and how the information is used in a Chinese context. There are likely to be differences among perceptions of health, telecommunication infrastructure, and patterns of inequalities in that of the West and East [16]. The increasing use of the Internet has raised important questions about the relationship between cultures and technologies.

Furthermore, scholars have repeatedly emphasized the need to give greater voice and influence to health consumers [3]. Much attention in the literature has focused on identifying who actively seeks or who does not seek health information, the frequency of use, and satisfaction with health information seeking [17-20]. There is a need for more qualitative research to examine how and why individuals obtain health information, where they go to retrieve such information, and how the health information is used. Qualitative research allows for greater exploration of reported behaviors in users. This allows for in-depth insights into the participants’ experiences, underlying motivations, and thoughts and feelings associated with health-seeking behaviors that are often not captured through quantitative methods [21,22]. Focus groups are commonly used in qualitative research as this method encourages interaction among participants, as well as free and open disclosure in a group context. It enables the researchers to have direct contact with key informants and allows researchers to gain substantive information in an easy and efficient manner [23]. Group interaction offers valuable data on the extent of consensus and diversity among the participants and allows the researcher to ask the participants themselves for comparisons among their experiences and views [23]. Health information seeking is a common shared experience, and thus, the use of focus group provides an opportunity for participants to interact and share rich sources of information that would otherwise not be obtained through individual interviews. Given the dearth of research in an Asian context, Hong Kong thus provides an excellent platform to explore individuals’ perceptions on health information seeking, where the use of the Internet and Web access devices is highly prevalent. This study, therefore, aimed to examine individuals’ perception on health information seeking and their related behaviors. This paper also reports on user’s perceptions of the advantages and disadvantages of seeking health information via the Internet and the application of the information obtained.

## Methods

### Study Design

Ethics approval was obtained from the institutional review board of the University of Hong Kong/Hospital Authority Hong Kong West Cluster. Five focus groups were conducted on Hong Kong adults from November 2015 to January 2016, with numbers in each group ranging from 9 to 11. Before recruitment, we proposed to conduct five focus groups based on recommendations from the literature on qualitative methods [24]—which allows flexibility in increasing or decreasing the number of focus groups after data collection has begun, and the focus group can stop when the point of data saturation is reached. Hence, we debriefed and reviewed the notes to reflect on each session before conducting the next session. By the fifth focus group, we were convinced that we had reached data saturation and stopped.

### Data Collection

This study was part of a larger research project entitled the Hong Kong Family and Health Information Trends Survey (FHInTS) that examined the general public opinions and behaviors on family health, information use, and health communication under the FAMILY Project at the School of Public Health, the University of Hong Kong. Details of the survey design have been reported elsewhere [25]. To facilitate the next phase of the research project, which included a random telephone-based household survey, focus groups were conducted to (1) obtain input and refine the survey and (2) elicit insights and perspectives on health information seeking. This paper reports on individuals’ perception on health information seeking and their related behaviors.

Recruitment of participants was conducted by the Public Opinion Programme (POP), a renowned survey agency in Hong Kong. Eligible participants were individuals residing in Hong Kong, aged 18 years or above, and fluent in Cantonese. A total of 3443 invitations were sent out to POP panel members either by email or phone (panel members were recruited through a consent question at the end of random telephone surveys. All members within a selected household were invited to enroll into the panel). Of the 82 participants who expressed interest, 49 participants participated.

Participation was voluntary, and written informed consent was obtained from the participants before the start of the focus group. Each focus group lasted for approximately 90 minutes and was managed by a panel of two members, specifically one moderator and one notetaker. An interview guide with prompts was developed to cover a range of key issues related to the research questions. Initially, five questions were asked:

1. What is your general perception on seeking health information online?
2. What are the benefits on seeking health information online?
3. What are the limitations on seeking health information online?
4. How do you search for information?
5. How is the information used?

After conducting the first focus group, transcripts and notes taken were reflected on before conducting the next one (including listening to audio record, reading notes, and debriefing with the research team); this resulted in an additional question for the subsequent sessions: (6) How do you use the health information obtained with your health professional?

The moderator was free to word and sequence questions in the most appropriate manner and to pursue areas in greater depth. Participants also completed a questionnaire on demographic characteristics. Participants who attended the focus group sessions were given HK $150 (US $1=HK $7.8) cash for their travel expenses and as a token of appreciation. Refreshments were made available.

### Data Analysis

All focus groups were conducted in Cantonese, audiotaped, and transcribed verbatim into Chinese by experienced researchers at POP. Reviewing the entire transcripts would represent a significant increase in costs and time. As recommended by Poland [26], a random small sample of transcript from each focus group was reviewed. This determined the extent to whether a full review of the rest of the transcripts was needed. A research assistant who was not involved in conducting the focus groups checked 10% of the transcripts of each focus group. No major errors were noted, and therefore, we were confident that the transcripts were of high quality. Thematic analysis [27] was used to identify, analyze, and report patterns (themes). First, the transcripts were read in detail, and broad themes were noted. Then an in-depth analysis was conducted using a process of constant comparisons in which differences and similarities were analyzed to identify main themes and subthemes. Another member of the research team and the first author (JTWC) cross-checked, discussed, and agreed on the coding of the data and confirmed that the themes identified reflected the data. In the event that researchers differed in their coding decisions, themes were reanalyzed and checked against other coded data until a consensus was reached. The transcriptions were in Chinese, and the analysis was conducted based on the Chinese transcript. Themes that emerged were later translated into English by the researcher who was bilingual and near-native in English and native in Chinese. Back translation was used for quality control check by an independent research assistant. This involved translating the English themes and quotes into the Chinese language. This ensured that the translated version reflects the item content of the original version. The quotes in the paper were from the English translation of the original Chinese transcripts.

## Results

### Participant Characteristics

The characteristics of the sample are shown in Table 1. The majority of participants were male (53.1%, 26/49) and married (51.1%, 25/49). A similar proportion was spread among the age groups. About half (51%, 25/49) had a tertiary education and were currently working, with 63.3% (31/49) earning more than the average household income of HK $20,200 (CSD, 2013).

Main themes and subthemes were identified and grouped into five categories. These include (1) Perceptions on seeking health information via the Internet, (2) Perceived benefits of the Internet, (3) Perceived limitations of the Internet, (4) Strategy to navigate the Internet for health information, and (5) Implications of seeking health information via the Internet. These are described below with translated quotations that attempted to preserve the intent of the speaker. The quotations were taken from a number of respondents and were identified based on groups (G1, G2, and so on), and participants (A, B, C, and so on). Multimedia Appendix 1 details the themes and subthemes identified.

### Perceptions on Seeking Health Information via the Internet

A majority (92%, 45/49) of respondents indicated that they did seek Web-based information and that they had sought health information via the Internet within the last 12 months. The main type of information that was sought includes healthy lifestyle advice (healthy eating and physical exercise) and prevention of chronic or infectious diseases. A majority (97%, 47/49) of these respondents agreed that the Internet was often the first medium they used to seek health information. As one respondent noted:

I think unless you are not familiar with the Internet, otherwise, it’s always the first place to go.

Group 3, Participant G

In regards to why they used the Internet, all respondents agreed that it was for the desire for greater understanding, clarity, and confirmation of the health issue. This was the case regardless of whether they were seeking information for themselves or for someone else.

**Table 1:** Demographic characteristics of the participants.

Characteristics		n (%)
**Sex (N=49)**		
	Men	26 (53)
	Women	23 (47)
**Age group, years (N=47)**^a^		
	18-24	9 (19)
	25-34	8 (17)
	35-44	8 (17)
	45-54	6 (13)
	55-64	8 (17)
	65+	8 (17)
**Education attainment (N=47)^a^**		
	Primary or below	2 (4)
	Secondary	14 (30)
	Tertiary or above	31 (66)
**Marital status (N=47)**^a^		
	Single	19 (40)
	Married	24 (51)
	Divorced or widowed	4 (9)
**Employment status (N=48)^b^**		
	Full-time	16 (33)
	Part-time	5 (10)
	Self-employed	5 (10)
	Unemployed	22 (46)
**Monthly household income (N=38)**^c^		
	<10,000	7 (18)
	10,000-19,999	7 (18)
	20,000-29,999	8 (21)
	30,000-39,999	4 (11)
	40,000+	12 (32)

^a^Missing n=2.

^b^Missing n=1.

^c^Missing n=11.

As noted by a male respondent:

My family members are elderly, I need to have some information first before persuading them to go see a doctor.

Group 2, Participant D

However, for a minority of respondents (8%, 4/49), particularly for those older than 55 years, traditional health services (eg, doctors and professionals) was a first point of call if they had a health problem. Only when traditional health services *failed* would they turn to the Internet to seek alternative treatment methods. Regardless of the severity of the health topic, older respondents preferred more traditional resources (eg, doctors and professionals, family and friends, printed newspapers, and radio or television) than the Internet. For example, a woman (aged 55+ years) noted that:

Amongst my friends, around our age, for us to actually seek online, it really has to be an illness that even the doctor or you have had it for a long time, sought help from a lot of places but still have not found a way to cure it. Then (you) may then search online and see if there is anything that might help.

Group 5, Participant I

One of the reasons for not seeking health information via the Internet was that it was difficult for respondents. Some (50%) of the older respondents claimed that they feel overwhelmed and nervous with using the Internet and that it was difficult to find information. Whereas some (50%) needed assistance to access Web-based information, others felt that a lot of effort was required to seek health information via the Internet:

I feel that [searching on the Internet] is bothersome. Sometimes when you search...I, myself, am not very good at searching. So going about it more directly (asking family/friends/professional) is faster.

Group 5, Participant I

### Perceived Benefits of the Internet

When asked about the benefits of seeking Web-based information, four common themes emerged. These included convenience, coverage of vast information, self-awareness, and being able to share experience and form support groups. Convenience was noted as the main benefit of seeking health information via the Internet and was agreed by all respondents. The convenience of Web-based health information encompassed the ease and speed of access, at any time, and from any location. This was contrasted with accessing traditional health services. For example, a female respondent noted that:

The Internet is really easy to use, you can use it anytime. Unlike doctors or health clinics, I can’t call them and ask them at work, and after work, they are all closed. But with the Internet, you can search the information during work, and even after work, you can use your mobile phone to go on the Internet to search. I think this is really convenient and because it’s the Internet, it offers you more sources and opinions.

Group 3, Participant G

Many (78%, 38/49) of the respondents (including those that do not seek information via the Internet) stressed their limited time in doctor consultations and the often lack of time to discuss or elaborate on certain issues. The Internet was thus perceived to be particularly useful for expanding on the information received from the doctor. An example was provided by a male respondent:

Often, doctors don’t have the time to speak with you at length, because if he/she gives you 15 minutes, it is already a lot. For example, with cancer, lymphoma, even if he/she has a report after you have been tested, I think if you make him/her explain for 5 minutes, it will be a painful process for him/her, right? Typically, professionals, they will usually tell you a few things, but not a detailed explanation, so it is necessary for me to rely on the Internet for however many hours (I need to understand the issue).

Group 3, Participant D

With the convenience of the Internet, respondents were also able to obtain a vast amount of information, and they often obtained more information than their initial search topic, thus allowing them to expand their knowledge. The ability to access a vast amount of information was noted by a majority (71%, 35/49) of the respondents as a perceived benefit. For example, a male participant noted:

When you search (on the Internet) you get a lot of related information. For example, if I was initially just searching for the cause of diabetes, there would also be links to diets for diabetes and other related issues. I probably never thought about these (related information) prior to searching the Internet.

Group 3, Participant F

For some (44%, 21/49), the Internet raised their awareness on certain health issues and allowed them to attend to their health problems early. Respondents, particularly younger participants (aged 18-35 years), felt that the Internet allowed them to become more active seekers for their own health. This was expressed by a female participant:

I think the best advantage of going online to look for information is that I take the initiative to go online to look for information. For example if I go to listen to a health lecture on health information, it is actually led by one speaker, the things he speaks about, are the things I absorb. But the Internet is as big as the world, I can pick and choose what I want, this is the advantage I feel, but of course it also has its disadvantages.

Group 4, Participant H

Female respondents (20%, 10/49), in particular, spoke of the Internet as a medium that allows individuals with similar health concern or background to share and support one another:

It is actually psychological support on some level, I feel, I am getting some support, and also I can actually see that [their condition] and [my condition] are similar, so “Oh, they are okay, so I should be okay too”, it is this kind of feeling.

Group 2, Participant K

### Perceived Limitations of the Internet

Despite the benefits, several limitations were noted by respondents about seeking Web-based health information. Specifically, trustworthiness, frustration and fear, and nontailored information were recurring themes that ran through all the focus groups. The quality and trustworthiness of the information on the Internet was a main concern for all of our respondents, particularly for those that did not seek information via the Internet (8%, 4/49). Even for young participants, finding credible health information via the Internet was not straight forward. As a male respondent noted:

I think there is too much information on the Internet, and sometimes you do not know if the information is right or wrong, so you need to read and know everything yourself, maybe it is easier to trust if you go to some more professional websites, right? With forums, maybe even believing 10% of it is problematic, so there is too much information, but it is very easy to search, but you need to filter it yourself. That means you still have to use some time to filter.

Group 4, Participant C

Respondents with lower education level attainment were more likely to report frustration during Web-based health information seeking. For example, the sheer volume of information was sometimes perceived as “daunting” and might cause confusion. A majority (71%, 35/49) of the respondents felt that the health information obtained could be misleading and may exaggerate the health problem, intensify anxious feelings, and delay seeking professional services. This frustration and fear from the sheer volume of health information applies to a range of health issues, including lifestyle information to life-threatening diseases:

There is so much information. For example, if I wanted information on healthy diet and how to lose weight, when you search, heaps and heaps of information comes up. So it’s really difficult to decide which to use, let alone whether it’s actually suitable for me or not, or even whether it’s trustworthy.

Group 2, Participant H

I think for some, if they need to have an operation and want to know more about it, they search the Internet. But some of the information regardless of it being true or not, may freak them out. They may delay having the operation and instead seek alternative non-traditional health services.

Group 3, Participant B

Furthermore, a couple of the respondents (31%, 15/49) felt that any advice provided over the Internet was limited by the fact that it was not based on the individual’s condition and knowledge of their past history:

Health information online is not tailored, so what works for one may not work for another, and I really don’t know whether it works for me.

Group 2, Participant J

### Strategy to Navigate the Internet for Health Information

A recurring theme that was identified through all the focus groups was the strategies employed in navigating Web-based health information. Almost all (97%) of the health information seekers began their search process with search engines. Google was the most common search engine used; however, most (51%) did not go beyond the first two pages of citations following the search:

For the search results from a Google search engine search, if you compare the first three to five results and they are mostly similar with few differences, then you will not want to go through the effort of reading the sixth.

Group 2, Participant J

A part of navigating the Internet involved how to determine what information or websites to use and trust. Choosing a credible website was regarded as a common sense activity; however, when asked about the details, respondents had trouble in articulating their selection process. Nevertheless, some respondents (31%, 15/49) were able to express sources of health information that they would not select. For example, respondents agreed that they tended not to trust corporate websites, specifically those of pharmaceutical companies or those that clearly advertise products. Respondents also reported looking for the country of origin of the information and had more confidence in websites from Taiwan or abroad than websites from China Mainland. They preferred information that originated from what they considered to be impartial and reputable sources such as government, professional, or disease-focused organizations, or university websites. Whereas all respondents agreed that the Hong Kong government websites were credible, all of them felt that it provided very little information:

You will have a look at where the site comes from, you will have a look at if its layout has a lot of games, you can feel that it is commercial...I tend to believe sites from Taiwan or abroad, and I do not really believe those from China, Mainland.

Group 2, Participant K

Respondents also noted that to further determine which health information is credible, information would be compared across several websites, and only when they appeared similar would respondents perceive it to be trustworthy:

I rarely search using Mainland Chinese websites, and also, I usually go to at least 4 or 5 sites, I usually do this, I only trust it if “Oh, they are similar”. I will not focus on one site, and completely trust it, I definitely will not.

Group 2, Participant B

### Implications of Seeking Health Information via the Internet

Respondents provided insights into using the health information obtained via the Internet for decision making. A number of respondents used the health information to understand a subject or topic better and/or to decide whether they needed to see a doctor and to determine what questions to ask their doctors. Specifically, 31% (15/49) respondents felt that the Internet allowed them to become informed users and be able to share decisions with their health professionals. For a majority (92%, 45/49) of the respondents, the doctor’s authority remains crucial and sometimes becomes even more important, as they sought clarification or understanding on the information gained from the Internet:

At least after reading [it online] yourself you know how to ask the doctor about it, if you do not read, then you will not know how to even begin asking.

Group 1, Participant A

For example as we said just now, if my arm is numb, I would not have known why before, but maybe now after going on the Internet and reading more, maybe whichever side of the brain has had a stroke, so I will immediately go to the doctor, and I will find out. Yes, you can say that [the Internet’s] preventive nature may actually increase the chances of me going to the doctor.

Group 5, Participant D

On the other hand, a minority (4%, 2/49) of the respondents expressed that they could use the information obtained to challenge the advice given by their health service providers. This challenge was an explicit response to not believing the health professionals:

The doctor isn’t always right, so you need to use the information (obtained from the Internet) to keep questioning them.

Group 5, Participant I

Regardless of the severity of the health issue, for respondents that did seek Web-based information, they agreed that the Internet was always the first source for information. In cases where the health concern was not perceived as severe, respondents preferred to self-manage based on the information obtained from the Internet. In cases where the concern was severe, traditional health service was still preferred. However, traditional service was often accessed after respondents had searched the Internet for information:

I feel that you need to have a look at the question of how important it is. For example, I might be very fat, need to lose weight, and need to maybe look at the calories in food, so I might go online, because these things are relatively not so harmful to myself, so it might be worth trusting. But if my kidney is really painful, my stomach is really painful, or such, I might go online and have a look at what the reasons are for these things, and then I will still go and consult a doctor.

Group 5, Participant D

Overall, information from the Internet was generally perceived to be supplementary material and that the Internet is not a replacement to accessing traditional health services:

Going on the Internet to look for health information, to me, is supplementary and auxiliary. Sometimes the doctor might not explain in enough detail, so [you] find some supplementary information on the Internet, it mainly performs an assisting function.

Group 3, G

## Discussion

### Principal Findings

The aim of this study was to gain a better understanding on individuals’ perceptions on health information seeking on the Internet and their related behaviors. The findings from a Chinese population highlight several important issues that could inform other rapidly developing regions with increasing Internet use.

Mirroring Western studies [28,29], younger respondents (18-45 years old) were more likely to use the Internet as a source for health information. Consistent with the literature, the Internet was valued for its convenience, breadth of information, and the capacity to provide peer support and social interaction [3]. Along with the reported advantages of the Internet, respondents also noted the inherent disadvantages (eg, credibility and sheer volume of information). The difficulty with navigating the Internet acted as a barrier for older respondents to seek Web-based health information.

Our findings align with the theory of planned behavior, which posits that intentions predict behavior, and intentions are in turn predicted by attitude. Generally, all of our respondents expressed the intention in knowing more about health issues. However, younger respondents tended to consult the Internet before seeking medical consultation. In this study, this was the case regardless of the severity of the health issue. Respondents attributed this tendency to seek Web-based information to the limited time of consultations they received from their doctors. On the contrary, doctor consultation remained as the first point of contact for older respondents. This behavior was mainly attributed to the paternalistic view that “doctors know best” and the distrust of the health information on the Internet. It’s also important to note that the Internet was viewed as a supplement to health care rather than a replacement for professional care by all of our respondents. Understanding individuals’ intentions and health information seeking behavior is important as it can assist in the development of recommendations and policies to guide more effective help seeking and self-management among individuals, leading to improve health outcomes. Our findings shed light on the critical belief that guided individuals, particularly the elderly, in the decision to engage in Web-based health-information seeking.

For some female respondents, the Internet further provided social support and reassurance on health issues. Previous studies have reported that patients’ feelings, psychological problems, families, social problems, expectations of their doctors, ideas about their illnesses, and fears are rarely discussed between the doctor and the patient [30]. Our findings suggest the potential of the Internet to offer support to a large group of health consumers where they can share their personal health and illness experiences; they can offer special insights and reflections from the lived experiences of their specific health conditions that doctors may not be able to provide.

It is worthy to note that the initial research questions did not focus on health information seeking and its related impact on the doctor-patient relationship. However, following the first focus group, it was apparent to us that this was an important aspect to consider, and we then added a new question. Previous studies in Hong Kong noted that doctors were more powerful in terms of medical treatment and advice, in which during consultation, patient autonomy and self-management of illness are not usually advocated [31]. Our findings, however, suggest a shift toward a more balanced relationship between the doctor and the patient. Young respondents, in particular, noted that health information obtained from the Internet allowed them to be more informed and able to share decisions with and question their health professionals. This is consistent with studies that have observed a more powerful and autonomous patient when one is equipped with more medical and health information [19,20]. This is particularly salient in a Chinese culture where there is often a hierarchy and power-imbalance between the doctor and patient. As individuals are feeling more empowered, and are more inclined toward being involved in their health and health decision making, it may impact and change the way in which individuals interact with their health professionals. Future research into the role of health information and the impact on doctor-patient relationship will be important as technology and patient demand continues to evolve.

All of our respondents were conscious that there was an abundance of poor-quality Web-based health information. Indeed, previous studies have raised concerns for the quality of health information on the Internet and noted that the potential harm from inaccurate health information sources may be significant [12,32]. Health information from unqualified sources may lead to inappropriate treatments or delays in seeking necessary health services [12]. It is therefore important to devise ways to help individuals to choose information that is informative, credible, and useful. Health professionals may also consider ways to introduce and discuss Web-based health information with their patients. This may alleviate concern about the quality and overwhelming information and could have a positive impact on the patient’s health care decisions and outcomes.

Our findings also suggest that although the Internet is an easily available source of health information, it may also create inequalities in health information accessibility, especially among the elderly, those with low income, and those with low educational attainment. It is important to note that the rapid advancement of technology can create a digital divide, where there are many individuals who do not possess the necessary skills or the devices needed to navigate the Internet and search for credible health information. We also observed that this group of individuals relied on traditional mode of health information delivery (ie, doctors). This is of concern, as these groups are those that are more likely to have health problems but are less likely to access health care services. This group may further be disadvantaged as health care providers are increasingly transitioning to digital and Internet technologies for disseminating health information. There is a need to consider how health information can be disseminated to this group of individuals.

### Limitations

Transferability of these findings is limited to populations similar to participants in this study. Although every effort was made to recruit a diverse sample, our sample was of higher socioeconomic status. Future studies are needed to examine the perceptions of those with lower socioeconomic status. Individuals younger than 18 years were not included in our sample. Given that children and adolescents are growing up fast in the digital age, understanding their perceptions may further our knowledge on their health information–seeking behaviors. Finally, certain behaviors appeared to be intuitive and were therefore difficult to articulate. For example, some of our respondents were not able to describe their search and appraisal processes. Observational strategies may need to be employed in future studies to examine how health information via the Internet is obtained and used.

### Conclusions

The rapid development of information technology (IT) has increased the importance and relevance of questions related to health information seeking via the Internet. Our study has revealed that older and less educated individuals were less likely to use the Internet to look for health information and had more challenges in benefiting from Web-based health information. We have also identified that the most predisposed to searching for health information on the Internet were motivated by limited doctor consultation time and barriers to accessing professional health services. Strategies for communicating and disseminating credible health information in a form that all users can understand and use are urgently needed. These include taking into account the variety of individual skills in both searching and critically evaluating information, as well as the skills to use digital devices and should reach those who prefer not to use the Internet for health information. Understanding how these skills—often referred to as digital health literacy—are related to adoption and usage of IT is necessary and should be useful for exploring health information needs in various socioeconomic groups. Studies on how seeking health information affect behaviors are also needed so that targeted interventions can be developed to improve health outcomes. The findings from the focus groups were used to fine-tune our FHInTS survey, which had since been developed, and data collection has been ongoing. Questions regarding the medium to access Web-based health information, credibility of information sources, possession of devices, access to health services, and demographics have all been refined or incorporated to provide a better understanding on health information–seeking behaviors among adults in Hong Kong. Due to the rapid technological and related behavioral changes, Web-based health information seeking and its effects need to be closely monitored.

## Appendix

Multimedia Appendix 1 Themes and subthemes from the focus groups (N=49).

## Abbreviations

CSD: Census and Statistics Department
FHInTS: Family and Health Information Trends Survey
HKMDS: Hong Kong Monthly Digest of Statistics
IT: information technology
POP: Public Opinion Programme
