# Supplementary material for: How, When and Why People Seek Health Information Online: Qualitative Study in Hong Kong
Source: Interact J Med Res. 2017 Dec 12;6(2):e24. doi: 10.2196/ijmr.7000 (PMC5743920; doi:10.2196/ijmr.7000)
Supplement: Multimedia Appendix 1 [file ijmr_v6i2e24_app1.pdf]

Supplementary table: Themes and subthemes from the focus groups (N=49)

Category, themes, and subtheme			Sample quotes
**Perceptions on seeking health information via the Internet—participants that seek web-based information (n=45)**			
	The Internet is the first place you go to (n=44)		Group 2, F: *At the beginning, because [the Internet] is a starting point.*
	**Reasons for seeking online**		
		Greater clarity (n=45)	Group 4, C: *When I want to know more about an issue, I go online to do find out more.*
		When you need information quickly (n=15)	Group 5, H: *Because it is faster, and compared to other methods, if you have to go to the library, then comparatively, it really uses more time, and also it is not necessarily...when it is really late [at night], I can still use the Internet.*
	**Type of information searched**		
		Healthy lifestyle advices (n=26)	Group 1, B: *I search for weight loss online, for example, what exercise to do, how many sets to do.*
		Prevention of chronic or infectious disease (n=17)	Group 2, K: *Like seasonal flu.*
**Perceptions on seeking health information via the Internet—participants that do not seek web-based information (n=4)**			

	Traditional resources as first point of call (n=4)		Group 5, E: *My family doctor knows all about me, so if I have any problems I will definitely contact him first. I am not saying that going online is not good, just that everyone's composition and problem is different, so my own view is like this.*<br><br>Group 5, I: *I either talk about it with friends, or go directly to ask the doctor.*
	**Reasons for not seeking via the Internet**		
		The Internet is overwhelming and difficult to find information (n=2)	Group 5, I: *I feel that [searching on the Internet] is bothersome. I, myself, am not very good at searching.*
		Needs assistance in accessing web-based information (n=2)	Group 5, E: *It's not easy to use, and I need my son to help me with it, so I may as well just ask him.*
**Perceived benefits of the Internet**			
	Convenience (n=36)		Group 4, A: *I can find information even when I am sitting at home, and do not need to take a long trek, or go to get it for some reason, whatever information you think of, you can get it off the Internet.*
	Vast amount of information (n=32)		Group 5, I: *Going online gives you broader results, if you ask about a certain thing, it will give you a lot of examples.*
	Self-awareness (n=20)		Group 3, 2: *I think it [the Internet] also increases awareness about your own health, like articles that pops up in your newsfeed, you sort of become aware of it and apply it to yourself.*

	Shared experience and support groups (n=9)		Group 2, B: *The Internet means that you can form or join certain groups that have similar circumstances, then that is actually good...For common health issues, they will feel that [groups are] very good in sharing experience like "Oh, after trying this thing it really does work".*
**Perceived limitations of the Internet**			
	Trustworthiness and misleading information (n=35)		Group 1, D: *You don't know whether [the information] is true or false.*
	Frustration and fear (n=21)		Group 5, E: *Sometimes when you know too much, you actually become very scared, I am like that.*
	Nontailored information (n=15)		Group 1, I: *[The information] might not be wrong, some certain websites might be right, but as it turns out it might not be right for everyone.*<br><br>Group 2, J: *There is a rather disadvantageous aspect to reading health information on the Internet which is that it is not very specific to the individual.*
**Strategies to navigate the Internet for health information**			
	Search engine (n=43)		Group 2, J: *You always start with a Google search.*
	**Determining credible information**		
		Reputable source (n=14)	Group 3, C: *The ones I mean are CEHU, Central Health Education Unit, they will put everything on there, looking on there should be the most reliable, they are produced by the government itself.*<br><br>Group 2, K: *You will have a look at where the site comes from, you*

			will have a look at if its layout has a lot of games, you can feel for yourself if that it is advertising.
		Comparing information across several websites (n=8)	Group 3, C: *You go down the list [of Google search] and you look at a few sites and see if they say the same thing.*
**Implications of seeking health information via the Internet**			
	Become informed users and help with decision making (n=14)		Group 1, D: *I think compare to thirty, forty years ago, we get a lot more information, and so we can actually talk to our doctors and ask them questions.*
	**Doctor-patient relationships**		
		Doctor authority remains crucial (n=41)	Group 3, E: *For very important [health issues], of course you can't just go online, doctors are still really important.*
		Information used to challenge health professionals (n=2)	Group 5, I: *The doctor isn't always right, so you need to use the information (obtained from the Internet) to keep questioning them.*
